# A dual MET/AXL small‐molecule inhibitor exerts efficacy against gastric carcinoma through killing cancer cells as well as modulating tumor microenvironment

**DOI:** 10.1002/mco2.11

**Published:** 2020-06-16

**Authors:** Chenjing Zhu, Huashan Shi, Min Wu, Xiawei Wei

**Affiliations:** ^1^ Laboratory of Aging Research and Cancer Drug Target State Key Laboratory of Biotherapy and Cancer Center National Clinical Research Center for Geriatrics West China Hospital Sichuan University Chengdu Sichuan China; ^2^ Department of Radiation Oncology Jiangsu Cancer Hospital & Jiangsu Institute of Cancer Research & The Affiliated Cancer Hospital of Nanjing Medical University Nanjing Jiangsu China; ^3^ Department of Biomedical Sciences School of Medicine and Health Sciences University of North Dakota Grand Forks North Dakota USA

**Keywords:** AXL, gastric cancer, inhibitor, LY2801653, MET

## Abstract

The receptor tyrosine kinases MET and AXL have been implicated in tumorigenesis and aggressiveness of multiple malignancies. We performed this study to evaluate the antitumor impact of LY2801653, a dual MET and AXL inhibitor on gastric cancer and to elucidate the underlying mechanisms. In the present study, tissue microarrays containing gastric cancer tissues were stained with MET and AXL antibodies, which showed the prognostic values of MET and AXL. Administration of LY2801653 inhibited cell proliferation, migration, epithelial‐mesenchymal transition, induced apoptosis, and cell cycle arrest. Xenograft mouse models showed suppressed cell proliferation of tumors in high MET and AXL expression cells. LY2801653 also inhibited the growth of MET and AXL‐independent cells at higher but clinically relevant doses through decreased angiogenesis and M2 macrophages in the tumor microenvironment. In conclusion, our study provides evidence for MET and AXL as prognostic biomarkers and potential therapeutic targets in gastric cancer. The dual MET/AXL inhibitor LY2801653 represents a promising therapeutic strategy for the treatment of patients with gastric carcinoma.

## 1 INTRODUCTION

Gastric cancer is one of the most common malignancies and the third leading cause of cancer‐related death in males in the developing world.^1^, ^2^ This disease accounts for more than 720 000 deaths annually, which imposes an enormous burden on society.^3^ Platinum‐based chemotherapy remains the standard treatment strategy for the treatment of gastric cancer; however, treatment efficacy is often countered by the development of resistance to chemotherapeutic agents, which contributes to the high rates of death.^4^ The identification of new prognostic and predictive biomarkers is needed to improve the outcomes of these patients.

In recent years, targeted therapies have been developed, such as HER2‐targeted trastuzumab, VEGF‐A‐targeted bevacizumab, and VEGFR‐2‐targeted ramucirumab.^2^, ^5^, ^6^, ^7^ These small molecular targeting drugs, when used as either a single agent or in combination with chemotherapy, were shown to significantly extend survival. Several studies have suggested the prognostic and predictive value of two receptor tyrosine kinases (RTKs), MET and AXL, as biomarkers and potential therapeutic targets in cancer treatment.^8^, ^9^, ^10^, ^11^

MET (also known as mesenchymal‐epithelial transition factor, or C‐MET) is a heterodimeric RTK activated by the hepatocyte growth factor (HGF, or scatter factor) ligand.^12^, ^13^ Binding to HGF leads to phosphorylation of the receptor and the subsequent activation of MET.^14^ MET activation induces multiple signaling cascades associated with cell proliferation, migration, invasion, survival, and branching morphogenesis.^15^, ^16^, ^17^ Dysregulation of the HGF/MET signaling axis has been described in a variety of malignant and premalignant lesions, including lung cancer, gastroesophageal cancer, hepatocellular carcinoma, breast cancer, ovarian cancer, multiple myeloma, and colorectal cancer^18^, ^19^, ^20^, ^21^, ^22^, ^23^, ^24^ and is associated with tumor growth, angiogenesis, invasion, and metastasis.^25^, ^26^ *MET* gene amplification has been reported in approximately 8% of patients with gastric cancer by fluorescence in situ hybridization (FISH) analysis,^8^, ^27^, ^28^, ^29^ while polymerase chain reaction (PCR)‐based assays showed that the occurrence of *MET* gene copy number increases to approximately 20%.^30^, ^31^, ^32^

AXL is a member of the tumor‐associated macrophage (TAM) subfamily that belongs to the RTKs. The TAM family is comprised of TYRO‐3, AXL, and MER.^33^ GAS6 serves as a ligand for AXL with a high binding affinity.^34^ GAS6/AXL signaling functions as an important pathway driving cancer cell survival, proliferation, migration, and invasion.^35^

MET and AXL activate common downstream signaling pathways, including PI3K/AKT and MAPK/ ERK networks,^12^, ^36^, ^37^ leading to tumor growth, metastasis, drug resistance, immune suppression, and the stem cell phenotype.^11^, ^35^, ^38^ In addition, both MET and AXL have been reported to be intrinsically linked to epithelial‐mesenchymal transition (EMT), promoting cell survival and cancer metastasis.^38^, ^39^, ^40^, ^41^, ^42^, ^43^ Previous studies have described different therapeutic approaches for inhibiting HGF/MET or GAS6/AXL signaling, such as small kinase inhibitors and monoclonal antibodies targeting MET and/or AXL.^12^, ^44^, ^45^, ^46^

In this study, we investigated the antitumor effects of LY2801653 (merestinib), an oral ATP‐competitive, dual MET, and AXL tyrosine kinase inhibitor, on gastric cancer. LY2801653 was reported to display both in vitro and in vivo inhibitory effects on cholangiocarcinoma and nonsmall cell lung cancer (NSCLC).^39^, ^47^, ^48^ A phase 1 clinical trial has already been completed regarding LY2801653 in patients with advanced cancer (NCT01285037) (results not reported for now). In our study, LY2801653 demonstrated potent antitumor effects on MET and AXL‐dependent MKN45 gastric cancer cells as well as MKN45‐derived xenograft models by killing tumor cells directly. In addition, it also inhibited the growth of MET and AXL‐independent SNU719 cells at higher but clinically relevant doses through tumor microenvironment. These findings indicate that the antitumor activities of LY2801653 could provide potential therapeutic applications in patients with gastric cancer.

## 2 RESULTS

### 2.1 MET and AXL expression in gastric cancer cell lines and tissues

**FIGURE 1:**
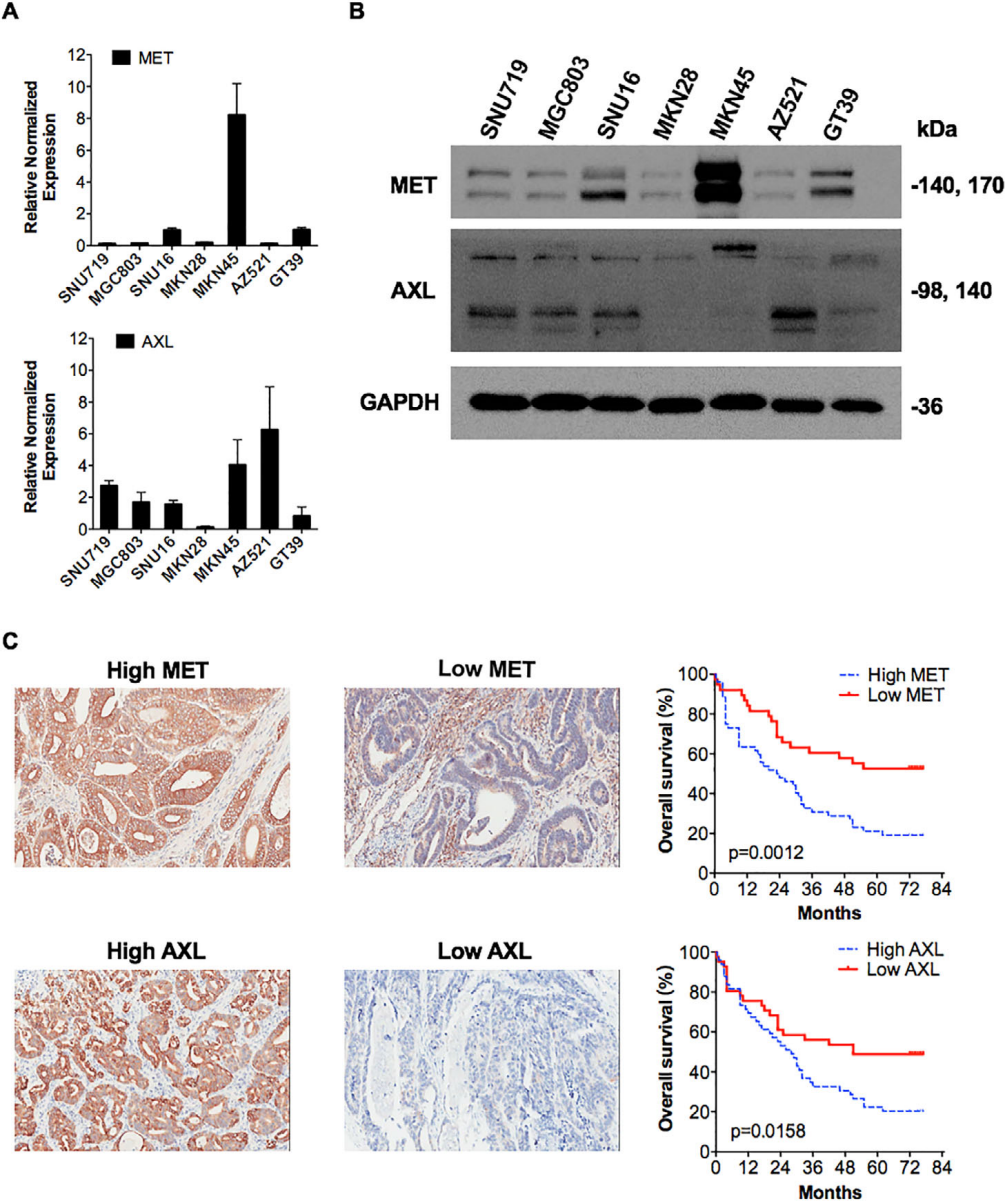
MET and AXL expression in human gastric cancer cell lines and gastric cancer patients’ tumors. Relative gene expression levels of MET and AXL in seven gastric cancer cell lines were analyzed using qRT‐PCR. Three cell lines (MKN45, SNU16, and GT39) showed overexpression of the MET gene, especially MKN45 cells. AXL expression was relatively lower in MKN28 and GT39 than in other cell lines. Data are presented as the mean ± SEM. All results are representative of three independent experiments. (B) Protein expression levels of MET and AXL were detected by Western blot analyses in seven gastric cancer cell lines. Prominent MET expression was observed in MKN45 cells. GAPDH was used as a loading control. (C) MET and AXL were differentially expressed in a 90‐gastric cancer tissue microarray. Representative images of low‐ and high‐MET‐ or AXL‐expressing microarrays are shown (20 ×). Both positively stained cells (0‐25% = 1, 26‐50% = 2, 51‐75% = 3, > 75% = 4) and the intensity of positive staining (negative = 0, weak = 1, moderate = 2, or strong = 3) were calculated. The multiplication of these two was the total score (values 0‐12), of which scores ≥6 defined high expression and < 6 defined low expression. High MET expression was associated with poor prognosis in patients with gastric adenocarcinoma (log‐rank *P* = 0.0012; HR = 2.37; 95% CI, 1.41‐3.89 for OS). Likewise, high AXL expression was related to short OS (log‐rank *P* = 0.0158; HR = 1.88; 95% CI, 1.14‐3.13)

We evaluated the expression of MET and AXL in a panel of seven gastric cancer cell lines by quantitative real‐time PCR (qPCR) and Western blot analyses. Three lines (MKN45, SNU16, and GT39) showed increased mRNA expression of *MET*. AXL expression was relatively lower in MKN28 and GT39 than in other cell lines. In particular, remarkable and prominent *MET* expression as well as high AXL expression was observed in MKN45 cells. Moderate MET and AXL expressions were seen in SNU719 cells (Figure 1A and 1B).

For gastric cancer patients, we calculated both positively stained cells (0‐25% = 1, 26‐50% = 2, 51‐75% = 3, > 75% = 4) and the intensity of positive staining (negative = 0, weak = 1, moderate = 2, or strong = 3) of tissue microarray. The multiplication of these two values resulted in the total score (values 0‐12), of which scores ≥6 were defined as high expression and those < 6 were defined as low expression. Pathologic analysis indicated that MET was expressed in 57.8% (52/90) of the gastric tissue microarray, and 42.2% (38/90) of the samples were negative for MET expression. AXL was expressed in 54.4% (49/90) of the microarray samples. Baseline characteristics of gastric cancer patients are presented in Table 1.

**TABLE 1:** Baseline characteristics of patients with gastric carcinoma

	Number	Percentage
Age		
Mean ± SD	67.43 ± 11.22 (95% CI: 65.08‐69.78)	
Gender		
Male	68	75.6
Female	21	23.3
Unknown	1	1.1
Histologic grade		
I‐ II	29	32.2
III	61	67.8
Clinical stage		
1	10	11.1
2	24	26.7
3	46	51.1
4	2	2.2
Unknown	8	8.9
T stage		
T1	4	4.4
T2	12	13.3
T3	42	46.7
T4a	11	12.2
T4b	7	7.8
Unknown	14	15.6
N stage		
N0	24	26.7
N1	14	15.6
N2	23	25.6
N3a	19	21.1
N3b	9	10.0
Unknown	1	1.1
M stage		
M0	88	97.8
M1	2	2.2

We further performed survival analyses to assess the prognostic value of MET and AXL in patients with gastric adenocarcinoma. Pathologic analysis of MET staining patterns indicated that MET expression was significantly associated with reduced overall survival (OS) (log‐rank *P* = 0.0012; hazard ratio (HR) = 2.37; 95% confidence interval (CI), 1.41‐3.89). In addition, we found that patients with high levels of AXL gene expression also had a significantly shorter OS (log‐rank *P* = 0.0158; HR = 1.88; 95% CI, 1.14‐3.13) (Figure 1C).

### 2.2 LY2801653 inhibited cell proliferation in gastric cancer cells harboring MET overexpression

**FIGURE 2:**
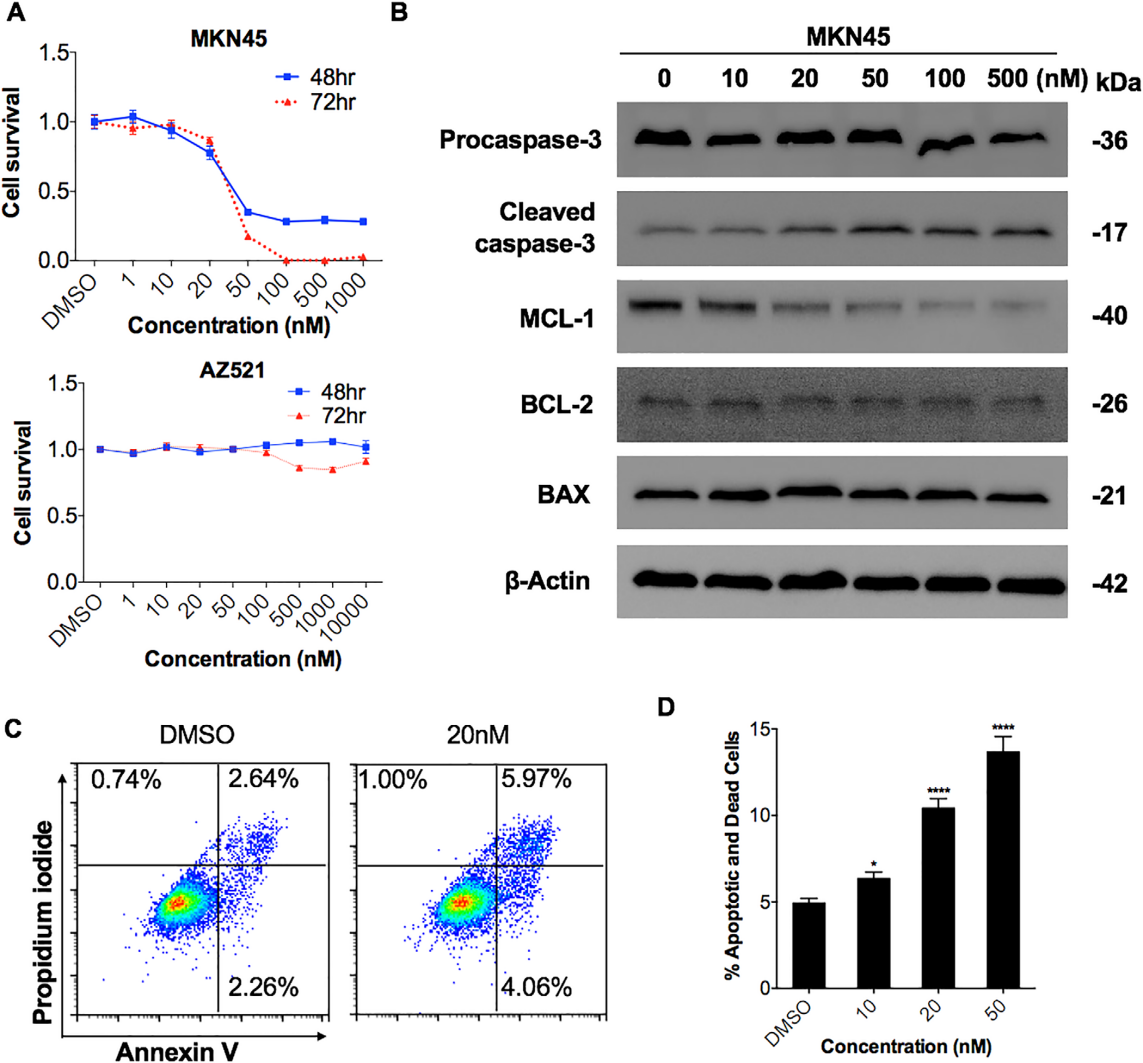
LY2801653 inhibited cell proliferation in MET‐overexpressing gastric cancer cells. (A) Cells were incubated with LY2801653 for 48‐72 h, and the effects of LY2801653 on the proliferation of gastric cancer cells were determined using a Cell Counting Kit‐8 (CCK‐8) assay. The IC50 of each treatment was calculated. Growth inhibition induced by LY2801653 was remarkable in *MET*‐amplified MKN45 cells with an estimated IC50 value of 24.83 nM (range: 20.85‐29.56 nM) for 48 h and 23.35 nM (range: 21.14‐25.8 nM) for 72 h, while other cell lines with low or moderate MET expression were not susceptible to increasing doses of LY2801653 (representative results of MKN45 and AZ521 cells under LY2801653 treatment are shown). (B) The effects of LY2801653 on the expression of apoptosis‐related proteins were detected by Western blot analysis. β‐Actin was used as an internal control. (C‐D) MKN45 cells were treated with either vehicle (DMSO) or LY2801653 (10, 20, and 50 nM) for 24 h, collected, stained with Annexin V‐FITC and PI, and analyzed by flow cytometry. Histograms of Annexin V and PI are shown. Early apoptotic cells are in the bottom right quadrant (Annexin V+/PI−), and late apoptotic or necrotic cells are in the top right and left quadrants (Annexin V/ PI+). The total levels of apoptotic cells, including early apoptotic, late apoptotic, and necrotic cells, were calculated. The levels of apoptotic cells significantly increased with LY2801653. All results are representative of three independent experiments. Data are shown as the mean ± SEM. ^*^ *P* < .05; ^**^ *P* < .01; ^***^ *P* < 0.001; ^****^ *P* < 0.0001; ns: not significant

A panel of seven cancer cell lines (MKN45, SNU719, MGC803, AZ521, GT39, SNU16, and MKN28) was incubated with increasing concentrations of LY2801653 (1‐10 000 nM) or vehicle for 48 or 72 h prior to performing proliferation assays. Dose‐dependent growth inhibition was measured by CCK‐8 assays. Overall, only *MET*‐amplified gastric cancer cells were susceptible to LY2801653 treatment. Growth inhibition induced by LY2801653 was prominent in MKN45 cells, with an estimated IC50 value of 24.83 nM (range: 20.85‐29.56 nM) for 48 h and 23.35 nM (range: 21.14‐25.8 nM) for 72 h. In contrast, the proliferation of other cell lines with low or moderate MET expression, even with high AXL expression such as AZ521 cells, was not significantly altered with increasing doses of LY2801653 (IC50 > 10 µM) (Figure 2A), indicating that MET might be a driver of cell proliferation.

### 2.3 LY2801653 induced apoptosis and cell cycle arrest in high MET and AXL expression gastric cancer cell lines

**FIGURE 3:**
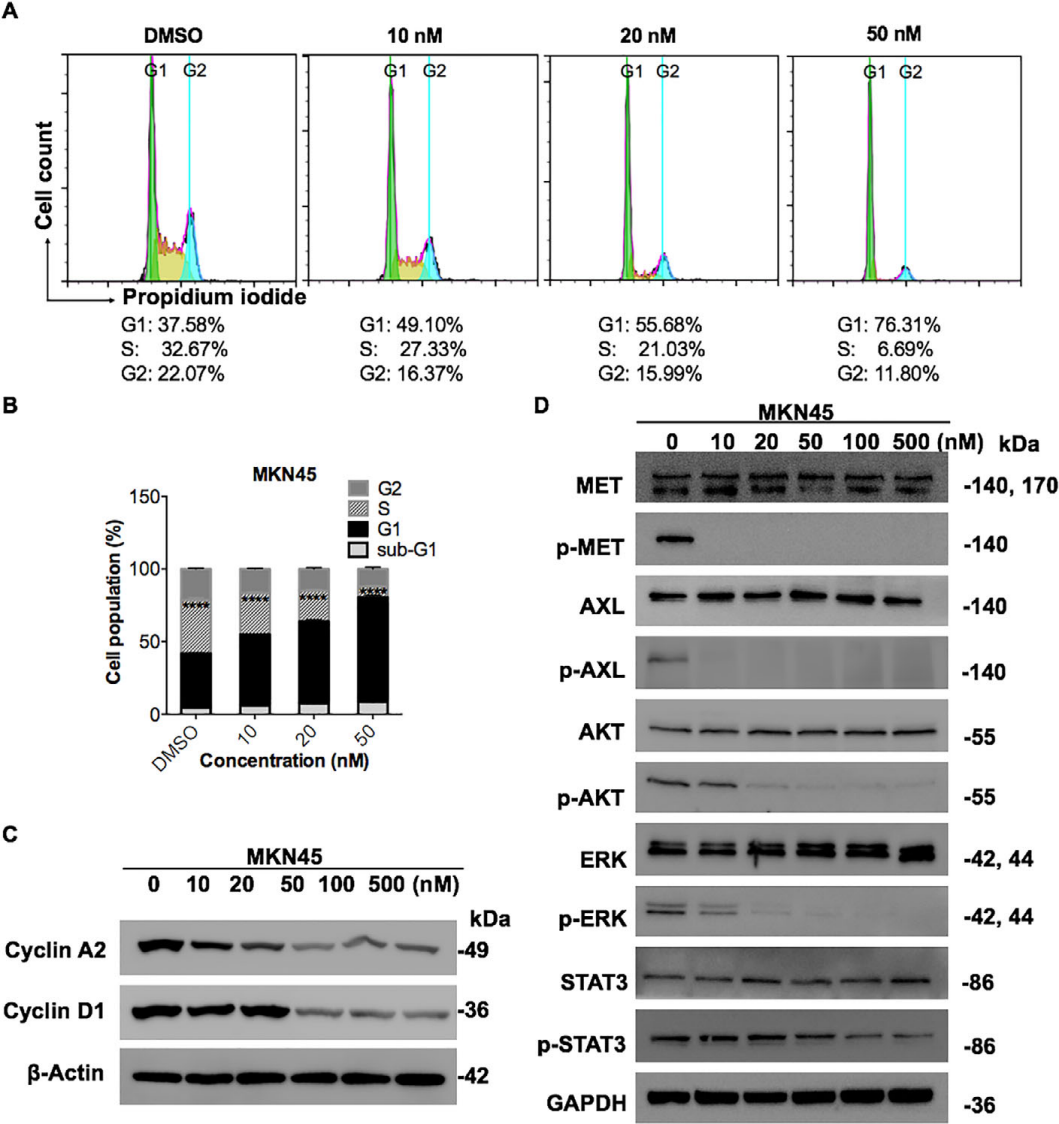
The MET/AXL inhibitor LY2801653 induced cell cycle arrest and inhibited downstream molecules in MKN45 cells. (A‐B) MKN45 cells were treated with either vehicle (DMSO) or increasing concentrations of LY2801653 (10, 20, and 50 nM) for 24 h, stained with PI (DNA content) and analyzed using flow cytometry. Cell cycle arrest at G1 phase was observed in MKN45 cells with lower proportions of cells in S phase as the doses of LY2801653 increased. Representative histograms are shown. All results are representative of three independent experiments. Data are shown as the mean ± SEM. ^*^ *P*  < .05; ^**^ *P* < 0.01; ^***^ *P* < 0.001; ^****^ *P* < 0.0001; ns: not significant. (C) Western blot analysis for Cyclin A2 and Cyclin D1 expression in MKN45 cells after serial diluted concentrations of LY2801653 treatment. β‐Actin was used as an internal control. (D) MKN45 cells were treated with the indicated doses of LY2801653 (0, 10, 20, 50, 100, 500 nM) for 24 h. Basal levels of MET, AXL, and their downstream molecules were determined by Western blot analysis. Data are representative of three independent experiments

We measured the effect of LY2801653 on the induction of apoptosis and cell cycle progression using flow cytometry. High MET and AXL expression MKN45 cells were treated with increasing concentrations of LY2801653 (10, 20, 50 nM) or diluent control for 24 h. The results showed a dose‐dependent increase in MKN45 cell death (early and late apoptosis/necrosis), either by Annexin V/PI assays (Figure 2C and 2D) or by cell cycle (sub‐G1) analysis (Figure 3B). In contrast, no significant effect on apoptosis was observed in low/moderate MET and AXL expression cells such as SNU719, even under 20 µM LY2801653 treatment compared to the DMSO group (Supplementary figure 1A and 1C). Western blot analysis confirmed an increased level of Cleaved caspase‐3 and a decreased level of MCL‐1 by concentrations of LY2801653 ranging from 10 to 500 nM, while Procaspase‐3, BCL‐2, and BAX remained unchanged (Figure 2B).

Additionally, upon incubation with LY2801653, high MET and AXL expression MKN45 cells had lower proportions of cells in S phase compared to the control group in a dose‐dependent manner, indicating cell cycle arrest at G1 phase (Figure 3A and 3B). Western blot analysis verified the diminished expression of the cell cycle regulatory factor Cyclin A2 and Cyclin D1 (Figure 3C), which is known to promote cell cycle G1/S transition.^49^ However, the effect was not obvious in gastric cancer cells with moderate MET and AXL expression SNU719 cells (Supplementary figure 1B and 1D).

### 2.4 Effects of LY2801653 on cell migration, EMT, and downstream signaling

**FIGURE 4:**
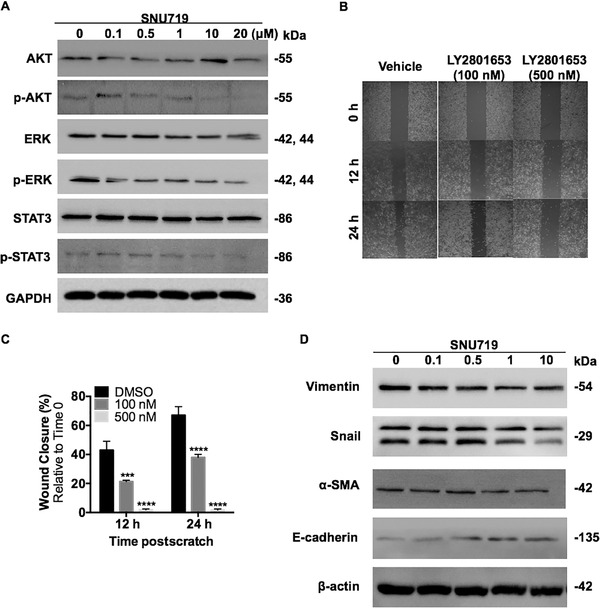
LY2801653 inhibited cell migration, EMT and downstream signaling in SNU719 cells. (A) SNU719 cells were treated with the indicated concentrations (0, 0.1, 0.5, 1, and 10 µM) of MET/AXL inhibitor LY2801653 for 72 h. Downstream proteins were evaluated by Western blot using specific antibodies. GAPDH was used as an internal control. (B‐C) SNU719 cells were treated with 100 nM, 500 nM LY2801653 or vehicle (DMSO), and subsequently subjected to scratch wound healing assay. Wound length was imaged and measured using imageJ after the wound was first made (0 h) and at the indicated time points (12 and 24 h) postwound exposure. Typical pictures of cell migration were shown (4 ×). Charts below shows that migration was significantly inhibited by 100 nM LY2801653 at 12 h (*P* < .001), and loss of migration ability was seen under 500 nM LY2801653 (*P* < .0001). Data are presented as mean ± SEM of three independent experiments, ^*^ *P* < .05; ^**^ *P* < 0.01; ^***^ *P* < 0.001; ^****^ *P* < 0.0001. (D) Western blot analysis of SNU719 cells showing the changes of EMT‐related proteins induced by the C‐MET/AXL inhibitor LY2801653. β‐Actin was used as an internal control

To assess the effect of LY2801653 on MET and AXL downstream signaling, high MET and AXL expression MKN45 cells were incubated with various doses of this agent (10‐500 nM) for a period of 24 h and evaluated by Western blot analysis. Treatment with LY2801653 at a concentration of 10 nM completely abolished MET and AXL autophosphorylation. A dose‐dependent inhibition of MET and AXL‐mediated effector molecules was observed, including p‐AKT, p‐ERK, and p‐STAT3, which were responsible for cell survival, cell proliferation, and transcriptional control, respectively (Figure 3D). The agent did not alter the total expression of MET, AXL, AKT, ERK, or STAT3, ruling out the possibility that variation in the phosphorylation status was the result of differential expression levels of proteins. Interestingly, SNU719 cells, which harbor moderate MET and AXL expression, were originally insensitive to LY2801653 under current treatment conditions. However, subsequent downstream signaling blockade was observed with the increasing of drug concentration (0.1‐20 µM) and action time (72 h) (Figure 4A).

Since MKN45 cells were semisuspended, we used adherent SNU719 cells to investigate the effect of LY2801653 on cell migration and EMT. SNU719 cells were treated with 100 nM, 500 nM LY2801653, or vehicle (DMSO) and subsequently subjected to scratch wound healing assay. We found that cell migration was significantly inhibited by 100 nM LY2801653 at 12 h (*P* < .001), and loss of migration ability was seen under 500 nM LY2801653 (*P* < .0001) (Figure 4B and 4C). Western Blot analysis showed the changes of EMT‐related proteins induced by increasing doses of LY2801653, including minimal impairment of Snail, Vimentin, and α‐SMA and induction of E‐cadherin protein expression (Figure 4D).

### 2.5 MET/AXL inhibition halted tumor growth in vivo with no obvious toxicities

**FIGURE 5:**
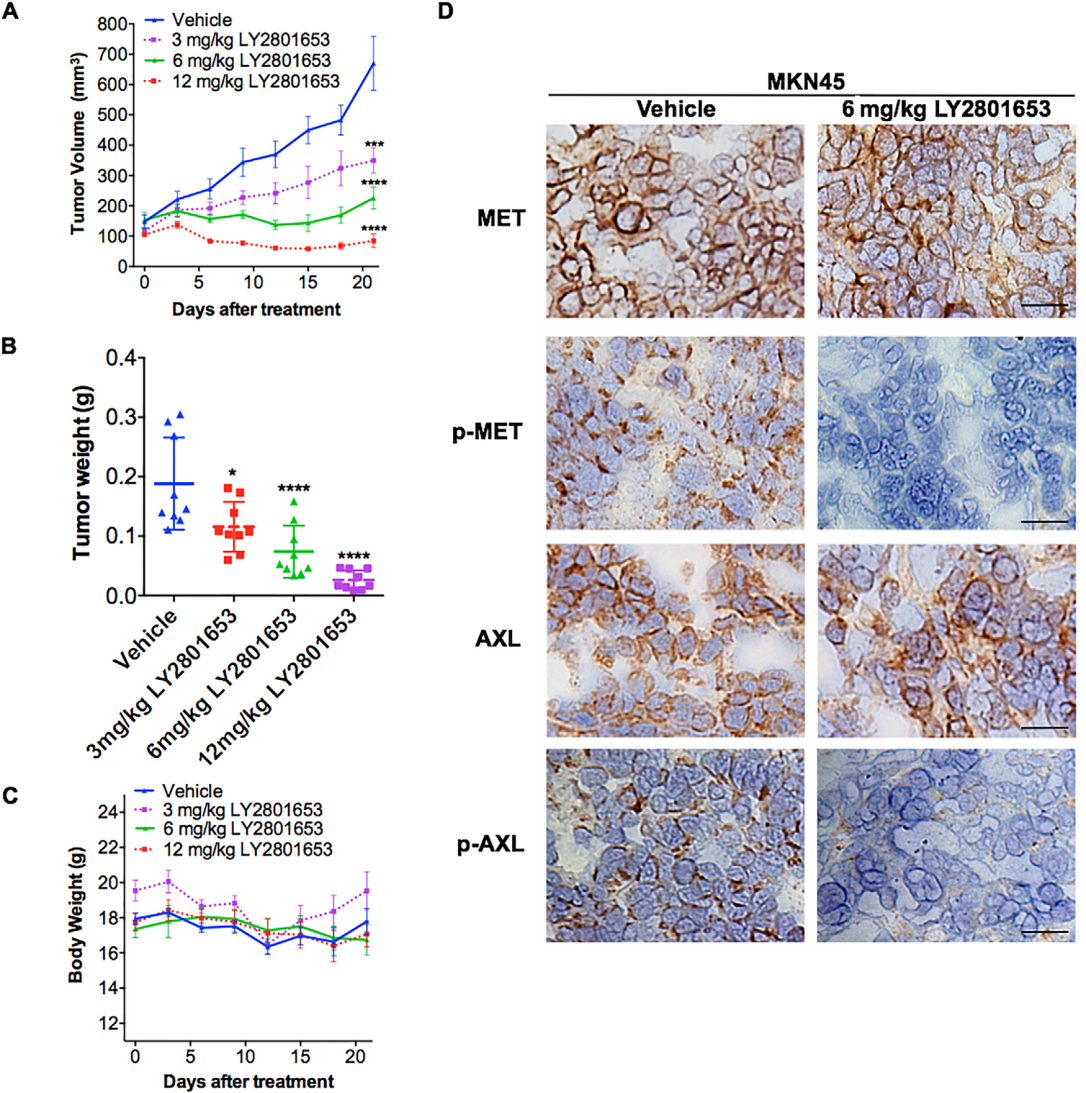
The MET/AXL inhibitor LY2801653 inhibits tumor growth of high MET and AXL expression MKN45 cells in vivo. (A) 1 × 10^7^ MKN45 cells were implanted subcutaneously into female Balb/c nude mice (6‐8 weeks). Once‐daily oral treatment with LY2801653 (3, 6, or 12 mg/kg) was given with vehicle solution as a negative control. Tumor length (*L*) and width (*W*) were measured with calipers every three days. TV was calculated as [TV = (*L* × *W* ^2^)/2], L: tumor length, and W: tumor width. Tumor volume was reported as the mean ± SEM. ^*^ *P* < 0.05; ^**^ *P* < 0.01; ^***^ *P* < 0.001; ^****^ *P* < 0.0001. *N* = 9 per group. (B) All xenograft tumor nodules from each mouse in the control group or treatment groups (3, 6, or 12 mg/kg LY2801653) were collected and weighed. Tumor weight was reported as the mean ± SEM. ^*^ *P* < 0.05; ^**^ *P* < 0.01; ^***^ *P* < 0.001; ^****^ *P* < 0.0001. *N* = 9 per group. (C) Body weight of each mouse throughout the experiment was measured. No significant difference in body weight was observed between vehicle‐treated mice (17.80 ± 0.74 g) and LY2801653‐treated mice (12 mg/kg: 17.08 ± 0.73 g; 6 mg/kg: 16.74 ± 0.86 g; 3 mg/kg: 19.53 ± 1.09 g) at the termination of the experiment (*P* > .05). (D) Xenograft tumors were stripped the day after the last treatment. Immunohistochemical analysis using MET, p‐MET (Tyr1234/1235), AXL and p‐AXL antibodies was performed in xenograft tumors treated with vehicle or 6 mg/kg LY2801653. Inhibition of p‐MET and p‐AXL staining was observed in the LY2801653‐treated xenograft tumors. Scale bar = 25 µm

To evaluate the effects of MET/AXL inhibition on the growth of gastric cancers in vivo, high MET and AXL expression MKN45 cells, as well as moderate MET and AXL expression SNU719 cells were used to establish gastric cancer xenograft models. Mice bearing established tumors were randomized into control and experimental groups, and treatment was initiated after the tumor volume reached a mean of 150‐200 mm^3^. A basal dose of 12 mg/kg LY2801653 was selected based on previous studies.^48^, ^50^, ^51^ The results showed a striking antitumor efficacy of LY2801653 against MET and AXL “high expression” model than “moderate expression” model. SNU719 tumors in the LY2801653‐treated group progressed more slowly compared to vehicle‐treated tumors (*P* < .05) at the termination of the experiment (Figure 7A). In MKN45‐bearing nude mice, a dose of only 3 mg/kg LY2801653 halted tumor growth significantly. On the last day of treatment, tumor volume differed between the control (670 ± 88.6 mm^3^), 12 mg/kg LY2801653 (84.27 ± 22.3 mm^3^) (*P* < .0001), 6 mg/kg LY2801653 (225.8 ± 35.57 mm^3^) (*P* < .0001), and 3 mg/kg LY2801653 (349.5 ± 41.43 mm^3^) (*P* < .001) groups (Figure 5A), which could also be reflected in tumor weight (Figure 5B). In addition, LY2801653‐treated mice showed virtually no weight loss throughout the experiment in both models (Figure 5C and 7B). Notably, no obvious abnormalities were found in hematoxylin‐eosin (HE) staining of the heart, liver, spleen, lung, and kidney of MKN45‐bearing nude mice in both the control group and LY2801653‐treated groups (*n* = 5 to 6 per group) (Supplementary figure 2A). Serum biochemistry analysis demonstrated no significant difference in fundamental biochemical indexes between the control and experimental groups (*P* > .05) except for 12 mg/kg LY2801653‐treated group which showed elevated liver enzymes (Supplementary figure 2B). In all, treatment with LY2801653 was well tolerated and effective.

### 2.6 Effect of LY2801653 on MET and AXL phosphorylation, angiogenesis, and tumor proliferation in the MKN45‐xenograft model

**FIGURE 6:**
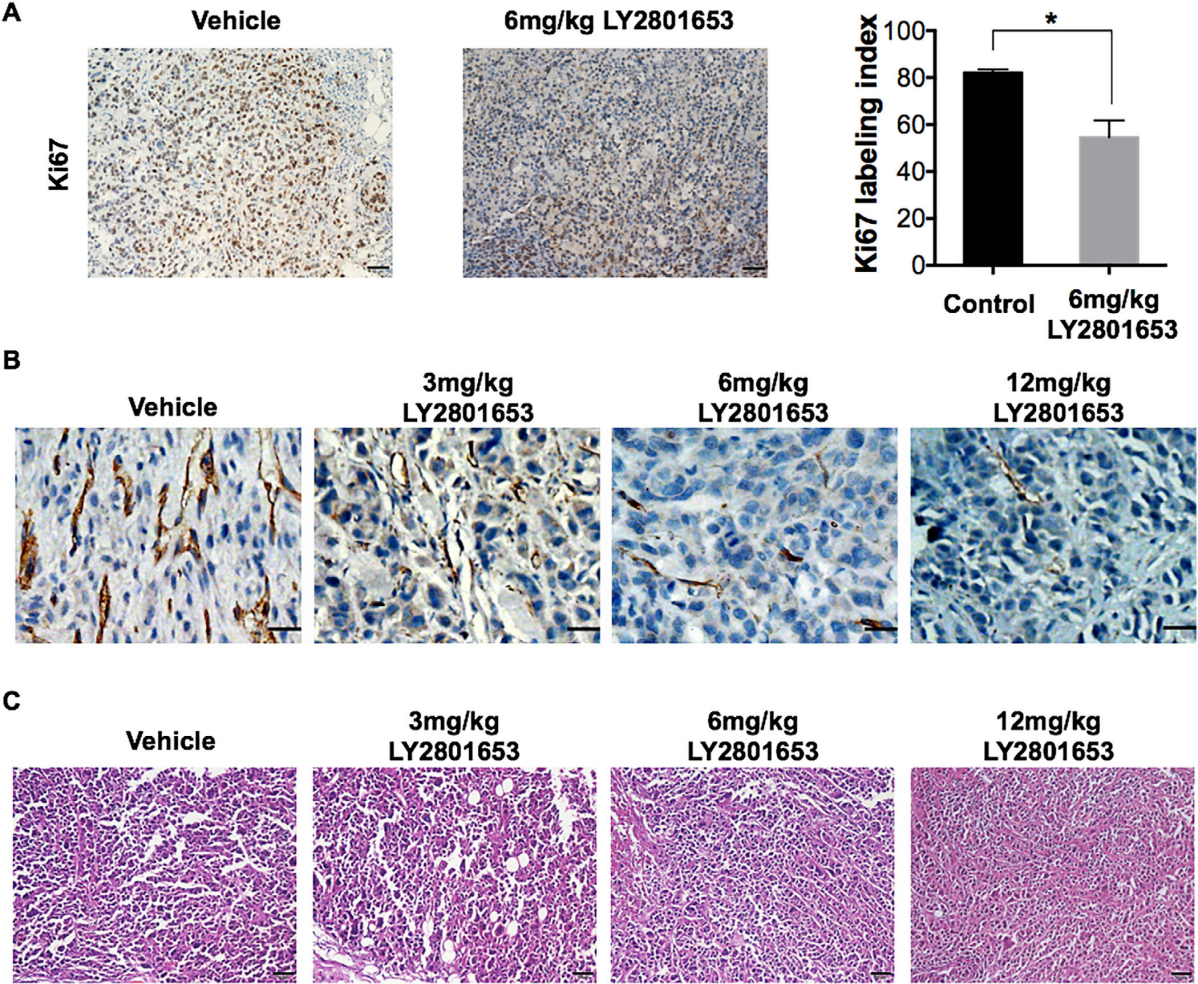
The MET/AXL inhibitor LY2801653 exerts antitumor effects through decreased proliferation and angiogenesis in MKN45 xenograft tumors. (A) Ki67 staining was performed for the control group and the 6 mg/kg LY2801653 treatment group. Immunopositive cells were counted in five arbitrarily selected fields, at 40× magnification in a blinded manner. The percentage of positive tumor cells was calculated as the Ki67 labeling index (LI). Treatment with LY2801653 significantly reduced the Ki67 LI in xenograft gastric tumor samples compared to the control (*P* < .001). Data are reported as the mean ± SEM. ^*^ *P* < 0.05; ^**^ *P* < 0.01; ^***^ *P* < 0.001; ^****^ *P* < 0.0001. Scale bar = 50 µm. (B) Tumor tissues in the control group and the 3, 6, or 12 mg/kg LY2801653 treatment group were stained with CD31. Treatment with LY2801653 decreased microvessel density compared to the control group with dose‐dependent effects. Scale bar = 25 µm. (C) Representative pictures of histopathology of xenograft tumors treated with LY2801653 or vehicle using HE staining under a light microscope (20×). Scale bar = 50 µm

Xenograft tissues were removed the day after the last dose of LY2801653, and we performed immunohistochemistry (IHC) to visualize the expression of MET and AXL and their downstream targets after LY2801653 treatment. Inhibition of both p‐MET and p‐AXL staining was observed in 6 mg/kg LY2801653‐treated MKN45‐xenograft (Figure 5D). A dose‐dependent attenuation of CD31 staining was observed, which implied decreased microvessel density in xenograft tumor tissues. Ki67 LI of gastric tumors in mice receiving LY2801653 (53.68 ± 2.51) was significantly lower than that in the control group (81.68 ± 4.32) (*P* < .001), demonstrating the induction of reduced proliferation in LY2801653‐treated tumors (Figure 6A‐C).

### 2.7 Effect of LY2801653 on tumor microenvironment

**FIGURE 7:**
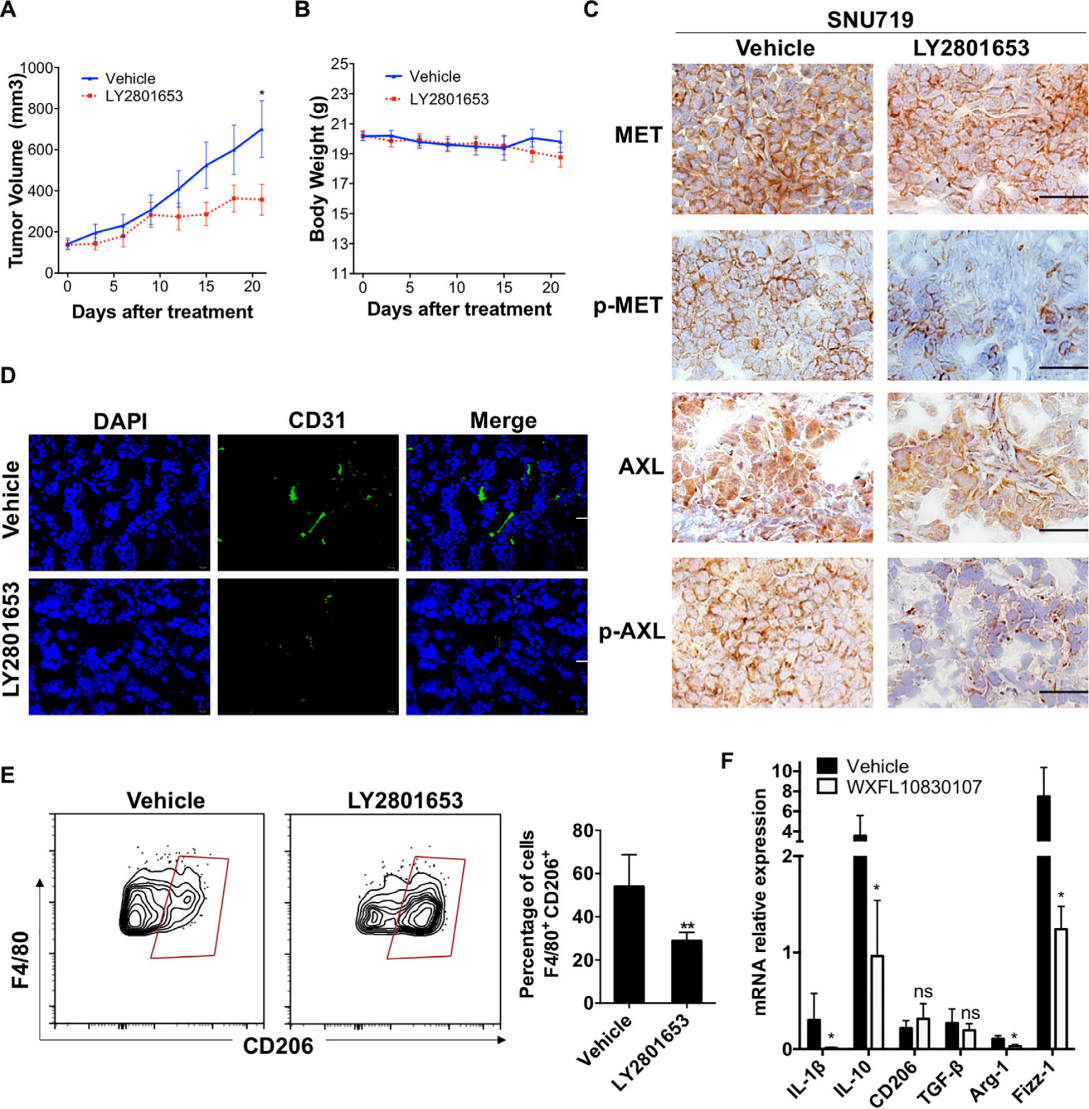
LY2801653 inhibited tumor growth of moderate MET and AXL expression SNU719 cells in vivo. (A) 1 × 107 SNU719 cells were implanted subcutaneously into female Balb/c nude mice (6‐8 weeks). Once‐daily oral treatment with LY2801653 (12 mg/kg, p.o.) was given with vehicle solution as negative control. Tumor length (*L*) and width (*W*) were measured with caliper every three days. Tumor volume (TV) was calculated as [TV = (L × W^2^)/2], L: Tumor length; W: tumor width. Tumor volume was reported as mean ± SEM. ^*^ *P* < 0.05; ^**^ *P* < 0.01; ^***^ *P* < 0.001; ^****^ *P* < 0.0001. *N* = 10 per group. (B) The body weight changes in different groups throughout the experiment. Treatment with LY2801653 was also well‐tolerated in SNU719‐bearing mice, with body weight for vehicle‐treated mice weighing 19.81 ± 0.11 g vs 19.59 ± 0.16 g in LY2801653‐treated group (*P* = .26) at the termination of the experiment. (C) LY2801653 inhibited p‐MET and p‐AXL expression in LY2801653‐treated xenograft tumors. (D) Representative photographs of CD31 immunofluorescence staining in the control group and 12 mg/kg LY2801653 treatment group. CD31‐positive cells were stained with FITC‐labeled antibody. DAPI‐stained nuclei were shown in blue. Merged pictures of CD31 and nuclei (right) showed cytoplasmatic colocalization of CD31. Treatment with LY2801653 decreased microvessel density compared to the control group. Scale bar = 20 µm. (E) The representative flow cytometry shows the expression of positive percentage of M2 (F4/80+ CD206+ cells, gated on CD45+) in xenograft tumors between LY2801653 and vehicle treatment groups (*n* = 5). Data are expressed as mean ± SEM and analyzed with Student's *t*‐test between two groups. ns, not significant, ^**^ *P* < 0.01. (F) mRNA expression of genes related to M2 tumor‐associated macrophages was detected by qPCR in SNU719‐derived xenografts. Compared to vehicle treatment, LY2801653 treatment of SNU719 tumor‐bearing mice decreases the production of immunosuppressive IL‐1β, IL‐10, Arg‐1, and Fizz‐1 in the tumor microenvironment (*n* = 5 per group). Data were reported as mean ± SEM. ^*^ *P* < 0.05; ^**^ *P* < 0.01; ^***^ *P* < 0.001; ^****^ *P* < 0.0001

The activity of LY2801653 against moderate MET and AXL expression gastric cancer xenograft model, SNU719, was also explored. IHC of xenograft tissues revealed inhibited MET and AXL activation (Figure 7C); however, in vivo tumor cell proliferation or apoptosis was not affected by LY2801653 (data not shown), which was in accordance with our in vitro results, suggesting the antitumor mechanism might be related to tumor microenvironment.

Apart from suppressed tumor angiogenesis (Figure 7D), we found that the percentage of M2 TAMs (CD45+ CD11b+ F4/80+ CD206+ cells) were significantly fewer in LY2801653 treatment group (28.82 ± 1.76) compared with control group (53.96 ± 6.62) (*P* < .01) (Figure 7E). qRT‐PCR of RNA from xenografts revealed a decrease in M2 markers in LY2801653‐treated tumors, including immunosuppressive IL‐1β, IL‐10, Arg‐1, and Fizz‐1 (Figure 7F). Other cells in the tumor microenvironment, such as myeloid‐derived suppressor cells, dendritic cells, neutrophils, and monocytes in xenograft tumors were not significantly changed between LY2801653 and vehicle treatment groups (data not shown).

## 3 DISCUSSION

Despite substantial improvements in therapeutic strategies, gastric cancer remains a major killer across the globe. Identification of specific prognostic biomarkers and drug treatable therapeutic targets is critical for gastric cancer management. The present findings established that both MET and AXL were independent predictors of gastric cancer prognosis. High MET expression was associated with poor prognosis with statistical significance based on the gastric cancer tissue microarray. Meanwhile, AXL could also serve as a survival predictor with detrimental OS in gastric cancer patients.

At present, the potential effects of MET and/or AXL targeting have been reported in renal cell carcinoma,^37^ lung cancer,^36^ and triple‐negative breast cancer.^52^ However, few reports are available regarding the combinatorial targeting of MET and AXL in gastric cancer. Our study demonstrated that the dual MET/AXL‐targeting compound LY2801653 has prominent antitumor activity at low doses for the treatment of high MET and AXL expression gastric cancer cells, LY2801653 inhibited proliferation, blocked phosphorylation of downstream AKT, ERK, and STAT3 proteins, and induced apoptosis and cell cycle arrest in high MET and AXL‐expressing MKN45 gastric cancer cells in vitro at nanomolar concentrations. In gastric cancer xenograft models, administration of LY2801653 significantly halted MKN45 tumor growth with inhibited MET and AXL phosphorylation activities, decreased cell proliferation and microvessel densities, and increased apoptosis in tumors. In addition, LY2801653 also inhibited the in vivo growth of MET and AXL‐independent cells at higher but clinically relevant doses through decreasing M2 macrophages in the tumor microenvironment. Taken together, our data indicated that the dual MET/AXL inhibitor LY2801653 successfully targeted MET and AXL by killing tumor cells or affecting tumor microenvironment, representing a promising strategy for the treatment of gastric cancer.

Both MET and AXL have been implicated in tumor cell survival, antiapoptosis, and tumorigenesis^13^, ^26^, ^36^, ^53^, ^54^, ^55^, ^56^; however, constitutively activated MET signaling is required for cell survival. *MET* gene amplification has been reported to exhibit constitutive ligand‐independent MET activation through receptor dimerization,^57^, ^58^ which acts as a primary “oncogenic driver” that might indicate poor prognosis in patients with gastric cancer.^28^, ^30^, ^31^, ^59^, ^60^ Smolen et al^61^ found that only a fraction of gastric cancer cell lines appeared to be extraordinarily vulnerable to the selective MET inhibitor PHA‐665752, and these cells were found to have high‐level amplification of wild‐type MET. In our study, analysis of a panel of seven gastric cancer cell lines using qPCR‐identified increased *MET* gene copy number only in MKN45 cells, which was further validated at the protein level by Western blot analysis. Treatment with the MET/AXL inhibitor LY2801653 led to a selective dramatic reduction in cell viability of MKN45 cells. In contrast, other cell lines were unaffected by the presence of LY2801653. Moreover, the induction of apoptosis and cell cycle arrest exclusively existed in MKN45 cells, which demonstrated the exquisite ability of MET to drive tumor cell proliferation and mitogenesis.

MET and AXL were shown to regulate cancer cell growth, migration, invasion, and drug resistance via altering MAPK/ERK, PI3K/AKT, and STAT3 signaling.^62^, ^63^, ^64^, ^65^ Our data suggested that inhibition of MET and AXL phosphorylation activities through LY2801653 in turn led to marked suppression of downstream oncogenic signaling pathways, which might play important roles in proliferation and angiogenesis‐related behaviors of cancer.

In tumor xenograft models, although a dose of 12 mg/kg LY2801653 has been shown to repress growth and progression in acute myeloid leukemia, gastric cancer, glioblastoma, pancreatic cancer, NSCLC, and colon cancer cells,^48^, ^51^, ^66^, ^67^ we found that a significant tumor growth delay was observed in high MET and AXL expression MKN45 gastric cancer model at a dose of only 3 mg/kg. LY2801653 showed robust antitumor activity by causing a reduction of proliferation and an increase in MKN45 cancer cell apoptosis. Moreover, LY2801653 did not directly eradicate tumor cells that moderately express MET and AXL, but act on tumor microenvironment. TAMs are a basic part of the tumor‐infiltrating immune cells which play an important role in immune system, leading to tumor escalation, angiogenesis, metastasis, and immunosuppression.^68^ In our study, we found that the immunoregulatory M2 macrophages, known to be involved in angiogenesis, tissue remodeling, and tumor progression,^69^ were significantly reduced after LY2801653 treatment. The underlying determinant of the in vivo sensitivity of SNU719 cells to LY2801653 remains under investigation.

## 4 CONCLUSIONS

In an era of personalized/precision medicine, searching for the right patient that best responds to certain RTK‐targeted therapies with tolerable adverse events is of utmost importance. Blockage of MET and AXL signaling might contribute to the antitumor effects in gastric cancer by inducing growth arrest and apoptosis in gastric cancer cell lines and suppressing cell growth in immunocompromised mice with no obvious toxicities. Our results suggested that LY2801653 might be a promising molecular inhibitory agent for combating human gastric cancers.

## 5 MATERIALS AND METHODS

### 5.1 Cell lines and reagents

Seven human gastric cancer cell lines, MKN45, SNU719, MGC803, AZ521, GT39, SNU16, and MKN28, were obtained from the American Type Culture Collection (ATCC). SNU719, MGC803, AZ521, GT39, and MKN28 cells were cultured in Dulbecco's modified Eagle's medium supplemented with 10% (v/v) fetal bovine serum (FBS) and 1% (w/v) penicillin‐streptomycin (Gibco). MKN45 and SNU16 cells were grown in RPMI 1640 supplemented with 10% (v/v) FBS and 1% (w/v) penicillin‐streptomycin (Gibco). All cells were cultured in a humidified incubator at 37°C in a 5% (v/v) CO_2_ atmosphere. All human cell lines were mycoplasma‐free and have been authenticated using short tandem repeat (or single nucleotide polymorphism) profiling within the last three years.

### 5.2 Antibodies and reagents

All antibodies used are indicated below: MET, phosphorylated MET (p‐MET) (Tyr1234/1235), phosphorylated AXL (p‐AXL) (Tyr702), p‐AKT (S473), STAT3, p‐STAT3, Procaspase‐3, Cleaved caspase‐3, BCL‐2, MCL‐1, Cyclin D1, E‐cadherin, Snail, α‐SMA, Vimentin, β‐Actin, GAPDH (Cell Signaling Technology, Inc., Danvers, MA, USA); AXL, AKT, ERK, phosphorylated ERK (p‐ERK), Ki67, CD31 (Abcam, Cambridge, UK); BAX and Cyclin A2 (HuaAn Biotechnology Co., Ltd., Hangzhou, China). LY2801653 was obtained from WuXi AppTec Group (Shanghai, China). For in vitro experiments, the compound was dissolved in DMSO (Sigma) to an initial concentration of 20 mM and further diluted to appropriate final concentrations in the relevant assay media. DMSO in the final solution did not exceed 0.1% (v/v). For in vivo studies, LY2801653 was formulated in 5% (v/v) DMSO, 40% (v/v) PEG‐400, 10% (v/v) solutol, and 45% (v/v) water and used at a concentration of 12, 6, and 3 mg/kg of body weight. Animals were treated with LY2801653 or vehicle control once daily by oral gavage.

### 5.3 Cell viability assay

Cell viability was determined using the Cell Counting Kit‐8 (Dojindo, Kumamoto, Japan) assay. Cells were seeded in a 96‐well plate at a density of 5000 cells/well. After seeding, cells were treated with vehicle or serial dilutions of LY2801653 for 48‐72 h. At the end of the experiments, 10% v/v CCK‐8 solution was added into each well per the manufacturer's protocol, and the cells were further incubated at 37 °C for 1  h. Absorbance was measured at 450 nm using a microplate reader. All experiments were performed in triplicate.

### 5.4 Western blot analysis

Cells were harvested and disrupted in a radioimmunoprecipitation assay (RIPA) lysis buffer (50 mM Tris (pH 7.4), 150 mM NaCl, 1% Triton X‐100, 1% sodium deoxycholate, 0.1% sodium dodecyl sulfate) with protease inhibitor cocktail. Equal amounts of whole cell lysates were resolved on a 10% sodium dodecyl sulfate–polyacrylamide gel electrophoresis (SDS–PAGE) gel (Bio‐Rad) and electrotransferred to a polyvinylidene difluoride membrane. The membrane was incubated with primary antibodies overnight at 4°C followed by incubation with secondary immunoglobulin G (IgG) and horseradish peroxidase linked antibody in TBS‐T for 1 h at room temperature. Enhanced chemiluminescent reagents were used to visualize the immunoreactive protein bands. The relative band density ratio was analyzed using ImageJ software (National Institutes of Health, Bethesda, MD, USA). The results were indicative of three independent studies.

### 5.5 Apoptosis assay and cell cycle analysis

Cells were collected with trypsin, washed three times with precooled PBS, resuspended in 300 µL of 1 × binding buffer, labeled with 2 µL FITC Annexin V, and 1 µL propidium iodide (PI) (BD Biosciences, CA) and analyzed by a NovoCyte Flow Cytometer (ACEA Biosciences, China).

For cell cycle analysis, cells were trypsinized, fixed, and then incubated with PI and RNase A (Sigma, St. Louis, MO, USA). Stained cells were then analyzed by flow cytometry. The percentage of cells distributed in the different cell cycle phases was quantified. Each experiment was repeated at least three times.

### 5.6 Quantitative real‐time PCR

The qPCR assay was carried out as described previously.^70^ In brief, total RNA was extracted using TRIzol (Invitrogen) according to the manufacturer's instructions. The RNA samples were then reverse‐transcribed to cDNA with the TaqMan Reverse Transcription Kit (Applied Biosystems, Foster City, CA, USA).

### 5.7 Scratch wound healing migration assay

Gastric cancer cells were plated in a six‐well plate. After overnight wounds were generated using a sterile 100 µL pipette tip. Cells were then exposed to specified treatments (vehicle or LY2801653). Multiple photographs of the wound were taken at 0, 12, and 24 h under an Olympus microscope at 4× magnification. The results were expressed as percentage of healing at indicated time points with respect to time 0 measured by ImageJ software.

### 5.8 In vivo studies

A total of 1 × 10^7^ gastric cancer cells were mixed with Matrigel (BD Biosciences) and implanted subcutaneously into female BALB/c nude mice (6‐8 weeks). The animal care was in accordance with institution guidelines. Once‐daily oral treatment with LY2801653 (12, 6, or 3 mg/kg) was given with vehicle solution as a negative control. Tumor length (*L*) and width (*W*) were measured with calipers every 3 days. Tumor volume (TV) was calculated as [TV = (*L* × *W* ^2^)/2]. At the end of the experimental period, mice were sacrificed by cervical decapitation, and the whole tumor was dissected, parts of which were used for HE, IHC, immunofluorescence staining and flow cytometry, and the rest was stored at −80°C for further studies. Vital organs of mice, including the heart, liver, spleen, lung, and kidney, were also harvested, paraffin‐embedded, and stained with HE.

### 5.9 Immunohistochemistry staining of xenograft tumor tissue and gastric cancer tissue microarray

For IHC analyses,^65^, ^71^, ^72^ we used 5 µm acetone‐fixed frozen xenograft tissue sections. Per the manufacturer's protocol, slides were labeled with primary antibodies. The secondary antibodies were biotinylated goat anti‐rabbit IgG. Images were taken with an Olympus microscope. Immunopositive cells were counted in five arbitrarily selected fields at 40× magnification in a blinded manner. The percentage of ki67‐positive tumor cells was then calculated as the Ki67 labeling index (LI).

The gastric cancer tissue microarray (Shanghai Outdo Biotech Co., Ltd.), which contained a total of 90 cases of gastric adenocarcinomas and their adjacent normal tissues, was stained for MET and AXL via IHC using the antibodies described above. Informed consent was obtained from all patients. Baseline levels of total MET and AXL were scored, quantified, and interpreted by two qualified pathologists using the Aperio imaging system (Leica).

### 5.10 Serum biochemistry analysis

The serum of mice in each group (*n* = 5–6) was obtained by centrifugation at the end of the experiment and used for serum biochemistry analysis with an automatic analyzer (Hitachi High‐Technologies Corp., Minato‐ku, Tokyo, Japan). Total bilirubin (TBIL), direct bilirubin (DBIL), alanine aminotransferase (ALT), aspartate aminotransferase (AST), total protein (TP), albumin (ALB), glucose (Glc), blood urea nitrogen (BUN), serum creatinine (S‐Cr), uric acid (UA), triglyceride (TG), total cholesterol (T‐CHO), high‐density lipoprotein (HDL), low density lipoproteins (LDL), alkaline phosphatase (ALP), CK‐MB, lactate dehydrogenase (LDH), and amylase (AMY) were evaluated for comparison between control and experimental groups.

### 5.11 Statistical analysis

Student's *t*‐test was used for data comparison between the control and experimental groups, and one‐way ANOVA was used to evaluate the difference among multiple groups. Statistical differences at *P *< .05 were considered significant.

## CONFLICTS OF INTEREST

The authors declare no potential conflicts of interest.

## ETHICAL APPROVAL AND CONSENT TO PARTICIPATE

All procedures performed in studies involving human participants were in accordance with the ethical standards of the institutional and/or national research committee and with the 1964 Helsinki declaration and its later amendments or comparable ethical standards. This study was approved by the Ethics committee of Shanghai Outdo Biotech Co., Ltd, China; all protocols for animal experimentation were approved by the State Key Laboratory of Biotherapy Animal Care and Use Committee of Sichuan University, China.

## AVAILABILITY OF DATA AND MATERIAL

The datasets used and/or analyzed during the current study are available from the corresponding author on reasonable request.

## AUTHORS/CONTRIBUTORS

WXW provided the idea and planned the study. ZCJ performed the experiments and wrote the article. WYQ performed the statistical analysis and helped with the final revision of the article. All authors reviewed the manuscript and approved the final manuscript.

## Supporting information

(A and C) SNU719 cells were treated with either vehicle (DMSO) or LY2801653 (0.5 µM, 1 µM, 10 µM or 20 µM) for 72 hrs, collected, stained with Annexin V‐FITC and PI, and analyzed by flow cytometry. The amounts of apoptotic cells did not change significantly under the effects of LY2801653. All results are representative of three independent experiments. Data are shown as mean ± SEM. ^*^ p < 0.05; ^**^ p < 0.01; ^***^ p < 0.001; ^****^ p < 0.0001; ns: not significant. (B and D) SNU719 cells were treated with either vehicle (DMSO) or increasing concentrations of LY2801653 (0.5 µM, 1 µM, 10 µM, or 20 µM) for 72 hrs, stained with PI (DNA content) and analyzed using flow cytometry. The effect of LY2801653 was not obvious in SNU719 cells. Representative histograms are shown above. All results are representative of three independent experiments. Data are shown as mean ± SEM. ^*^ p < 0.05; ^**^ p < 0.01; ^***^ p < 0.001; ^****^ p < 0.0001; ns: not significant.

(A) Haematoxylin‐eosin staining of vital organs of MKN45‐implanted nude mice in both the control group and the LY2801653‐treated group (n = 5–6 per group). Several scattered bleeding points in the myocardium and lung were found in the LY2801653 experimental group and control group, which may be caused by the execution of mice or anatomical manipulation. No obvious abnormalities were found. Scale bars = 100 µm. (B) Serum biochemistry analysis was carried out in the control group and LY2801653‐treated groups (n = 5–6 per group). No statistically significant differences in fundamental biochemical indexes between the control, 3 mg/kg and 6 mg/kg experimental groups (p > 0.05) were found. However, TBIL, DBIL, ALT, ALP, LDH and CKMB was seen elevated in 12 mg/kg LY2801653‐treated group. Abbreviations: Total bilirubin (TBIL), direct bilirubin (DBIL), alanine aminotransferase (ALT), aspartate aminotransferase (AST), total protein (TP), albumin (ALB), glucose (Glc), blood urea nitrogen (BUN), serum creatinine (S‐Cr), uric acid (UA), triglyceride (TG), total cholesterol (T‐CHO), high‐density lipoprotein (HDL), low density lipoproteins (LDL), alkaline phosphatase (ALP), CK‐MB, lactate dehydrogenase (LDH), amylase (AMY). Data are reported as the mean ± SEM.
