# Supplementary figures and images for: A dual MET/AXL small‐molecule inhibitor exerts efficacy against gastric carcinoma through killing cancer cells as well as modulating tumor microenvironment
Source: MedComm (2020). 2020 Jun 16;1(1):103–18. doi: 10.1002/mco2.11 (PMC8489669; doi:10.1002/mco2.11)

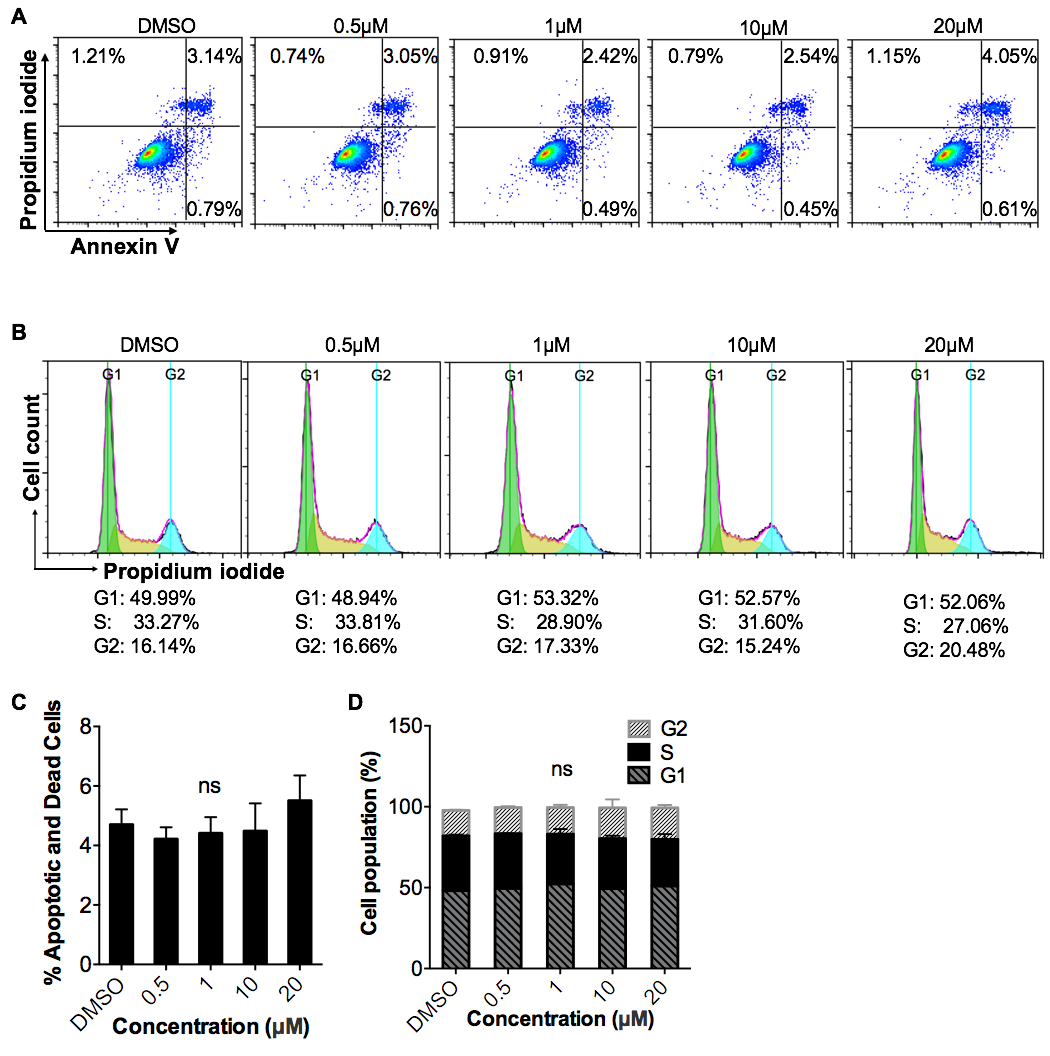

Supplement: Supplementary file 1 — (A and C) SNU719 cells were treated with either vehicle (DMSO) or LY2801653 (0.5 µM, 1 µM, 10 µM or 20 µM) for 72 hrs, collected, stained with Annexin V‐FITC and PI, and analyzed by flow cytometry. The amounts of apoptotic cells did not change significantly under the effects of LY2801653. All results are representative of three independent experiments. Data are shown as mean ± SEM. * p < 0.05; ** p < 0.01; *** p < 0.001; **** p < 0.0001; ns: not significant. (B and D) SNU719 cells were treated with either vehicle (DMSO) or increasing concentrations of LY2801653 (0.5 µM, 1 µM, 10 µM, or 20 µM) for 72 hrs, stained with PI (DNA content) and analyzed using flow cytometry. The effect of LY2801653 was not obvious in SNU719 cells. Representative histograms are shown above. All results are representative of three independent experiments. Data are shown as mean ± SEM. * p < 0.05; ** p < 0.01; *** p < 0.001; **** p < 0.0001; ns: not significant. [file MCO2-1-103-s002.png]

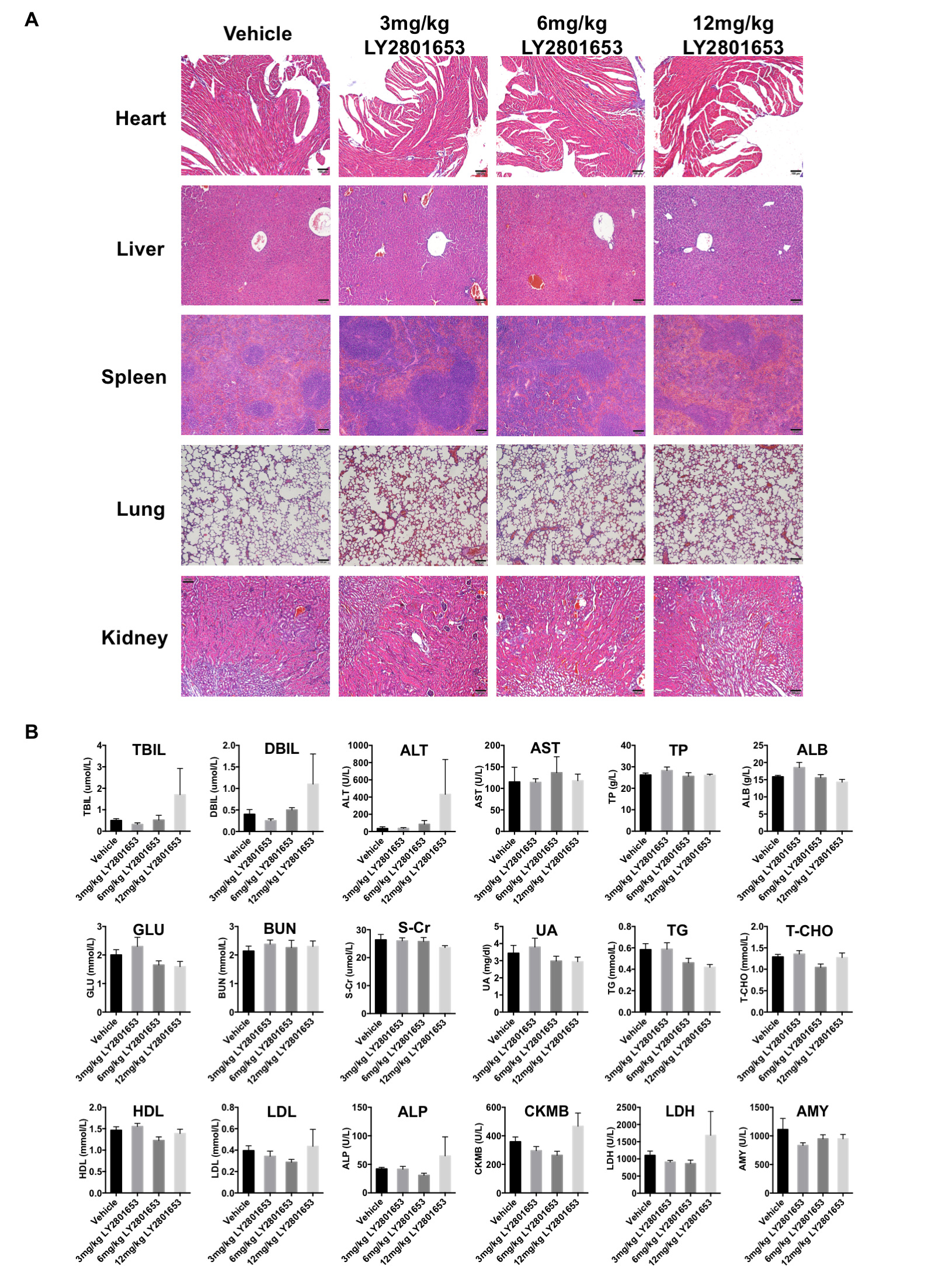

Supplement: Supplementary file 2 — (A) Haematoxylin‐eosin staining of vital organs of MKN45‐implanted nude mice in both the control group and the LY2801653‐treated group (n = 5–6 per group). Several scattered bleeding points in the myocardium and lung were found in the LY2801653 experimental group and control group, which may be caused by the execution of mice or anatomical manipulation. No obvious abnormalities were found. Scale bars = 100 µm. (B) Serum biochemistry analysis was carried out in the control group and LY2801653‐treated groups (n = 5–6 per group). No statistically significant differences in fundamental biochemical indexes between the control, 3 mg/kg and 6 mg/kg experimental groups (p > 0.05) were found. However, TBIL, DBIL, ALT, ALP, LDH and CKMB was seen elevated in 12 mg/kg LY2801653‐treated group. Abbreviations: Total bilirubin (TBIL), direct bilirubin (DBIL), alanine aminotransferase (ALT), aspartate aminotransferase (AST), total protein (TP), albumin (ALB), glucose (Glc), blood urea nitrogen (BUN), serum creatinine (S‐Cr), uric acid (UA), triglyceride (TG), total cholesterol (T‐CHO), high‐density lipoprotein (HDL), low density lipoproteins (LDL), alkaline phosphatase (ALP), CK‐MB, lactate dehydrogenase (LDH), amylase (AMY). Data are reported as the mean ± SEM. [file MCO2-1-103-s001.tiff]
